# Supplementary material for: Patterns of Intron Gain and Loss in Fungi
Source: PLoS Biol. 2004 Nov 30;2(12):e422. doi: 10.1371/journal.pbio.0020422 (PMC532390; doi:10.1371/journal.pbio.0020422)
Supplement: Table S1 — Also available at http://genes.mit.edu/NielsenEtAl/. (4.3 MB ZIP). [file pbio.0020422.st001.zip › NielsenEtAl/html/1407.html]

AN4202.1.NCU01221.1.MG05449.1.FG02099.1


```
 CLUSTAL W (1.82) Multiple Sequence Alignments - Introns Inserted


Sequence 1: AN4202.1	202 aa
Sequence 2: FG02099.1	202 aa
Sequence 3: NCU01221.1	202 aa
Sequence 4: MG05449.1	202 aa
Alignment Length: 202 aa
Number Identitical Residues: 159 aa
Alignment Score (without introns) 6572


MG05449.1 	MSSFESV0VVIDGKGHLLGRLASIVAKQLLEGQKIVVVRCEALNISGEFFRAKL1KYHAH
NCU01221.1	MSSFESV0VVIDGKGHLLGRLASIVAKQLLNGQKIVVVRCEALNISGEFFRAKL1KYHSY
FG02099.1 	MSSFEQV0VVIDGKGHLLGRLASIVAKQLLSGQKIVIVRCEALNISGEFFRAKL1KYHAH
AN4202.1  	MSSIDPV0VVIDGKGHLLGRLASTVAKQLLNGQKIVVVRCEALNISGEFFRAKL1KYHAY
          	***:: * *************** ******.*****:***************** ***::

MG05449.1 	LRKITRYNPTRGG~PFHFRAPSRVFYKAVRGMMPHKTARGTAALERLKVFEGVPPPYDKQ
NCU01221.1	LRKMTRYNPTRGG1PFHFRAPSRIFYKAVRGMIPHKTARGAAALERLKVFEGVPPPYDKK
FG02099.1 	LRKITRYNPTRGG1PFHFRAPSRIFYKAVRGMIPHKTARGAAALERLKVFEGVPPPYDKT
AN4202.1  	LRKITRYNPTRGG~PFHFRAPSRIFYKAVRGMIPHKTARGAAALERLKVFEGVPPPYDKK
          	***:********* *********:********:*******:****************** 

MG05449.1 	KKMVVPQALRVLRLQPGRKYCTVGRLSHEVGWKYQDVVAR2LEERRKAKAGAYYERKKLV
NCU01221.1	KKMVVPQALRVLRLQPGRKFCTVGRLSSEVGWKYEDVVSR2LEERRKAKGAAYYERKKVA
FG02099.1 	KKVVVPQALRVLRLQPGRKFCTVGRLSHEVGWKYQDVVAR2LEERRKAKGAAYYERKKIA
AN4202.1  	KRVVVPQALRVLRLRPGRKYCTVGRLSHEVGWKYQDVVAR2LEERRKVKSSAYYERKKAA
          	*::***********:****:******* ******:***:* ******.*..******* .

MG05449.1 	ARQRSESKKTANVDKKTAETLQAYGY
NCU01221.1	ARQLSEAKKSAKVNDKTAEALKEFGY
FG02099.1 	ARQLADAKKNATVKEETSKALANYGY
AN4202.1  	RRQLAHAQKSASVPENTKSQLAEYGF
          	 ** :.::*.*.* .:* . *  :*:
```
